# The Refinement of Ipsilateral Eye Retinotopic Maps Is Increased by Removing the Dominant Contralateral Eye in Adult Mice

**DOI:** 10.1371/journal.pone.0009925

**Published:** 2010-03-29

**Authors:** Spencer L. Smith, Joshua T. Trachtenberg

**Affiliations:** Department of Neurobiology, University of California Los Angeles, Los Angeles, California, United States of America; Ludwig Maximilians University Munich, Germany

## Abstract

**Background:**

Shortly after eye opening, initially disorganized visual cortex circuitry is rapidly refined to form smooth retinotopic maps. This process asymptotes long before adulthood, but it is unknown whether further refinement is possible. Prior work from our lab has shown that the retinotopic map of the non-dominant ipsilateral eye develops faster when the dominant contralateral eye is removed. We examined whether input from the contralateral eye might also limit the ultimate refinement of the ipsilateral eye retinotopic map in adults. In addition, we examined whether the increased refinement involved the recruitment of adjacent cortical area.

**Methodology/Principal Findings:**

By surgically implanting a chronic optical window over visual cortex in mice, we repeatedly measured the degree of retinotopic map refinement using quantitative intrinsic signal optical imaging over four weeks. We removed the contralateral eye and observed that the retinotopic map for the ipsilateral eye was further refined and the maximum magnitude of response increased. However, these changes were not accompanied by an increase in the area of responsive cortex.

**Conclusions/Significance:**

Since the retinotopic map was functionally refined to a greater degree without taking over adjacent cortical area, we conclude that input from the contralateral eye limits the normal refinement of visual cortical circuitry in mice. These findings suggest that the refinement capacity of cortical circuitry is normally saturated.

## Introduction

In development, initially promiscuous synaptic connections are refined by experience-dependent plasticity processes into an exquisite, highly functional circuit [1]–[3]. Perhaps the most fundamental feature of visual cortical circuitry is retinotopy. The refinement of retinotopic maps proceeds rapidly following eye opening and asymptotes during maturity, but it is unknown what limits the refinement.

One possibility is that the quality of the sensory input fails to support further plasticity processes. If this is true, then it implies that there may be latent capacity in the circuitry for further refinement given changes in the sensory input. Alternatively, it could be the case that the refinement is terminated once the circuitry cannot support any further refinement. For example, perhaps inputs from the two eyes compete for circuitry through synaptic plasticity mechanisms, as seen in the precritical period [2]. If this is true, then if activity from one of the two eyes was terminated, further refinement of the remaining eye's retinotopic map would be possible using the same population of neurons.

Interestingly, human [4]–[6] and rodent [7]–[8] studies have shown that behaviorally assessed single eye acuity increases following monocular deprivation or loss of function of the fellow eye. Although it is unknown where the physiological changes underlying this effect take place, it remains possible that one contributing locus is primary visual cortex, where information from the two eyes is first integrated.

**Figure 1:**
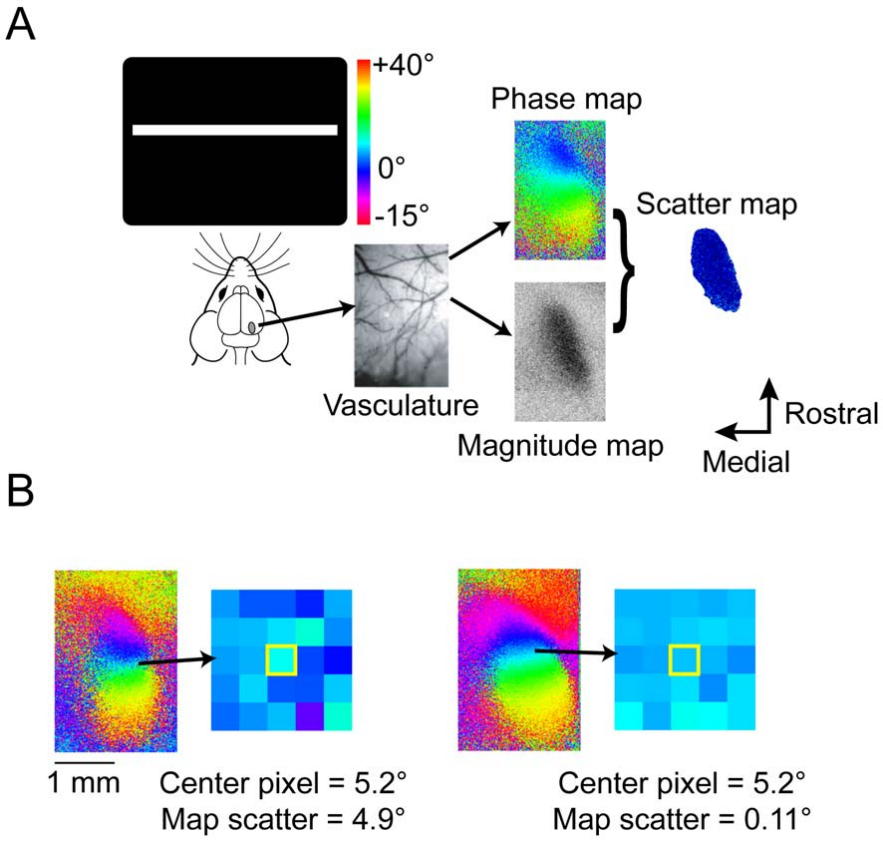
Scatter measures the quality of retinotopy and values decrease in development. (A) The visual cortex was imaged while the mouse watched a white bar drift across the stimulus screen. The evoked intrinsic signal was optically imaged by measuring the reflectance of 700 nm light off of the brain. Using Fourier analysis, magnitude and phase response maps were computed from the image data and used to generate a scatter map. (B) Scatter is measured as the difference, in degrees of elevation in visual space, between a pixel and its neighbors in a 5×5 box. Pixels from two different ipsilateral eye retinotopic maps (from a P17 mouse at left, and a P21 mouse at right) have the same receptive field elevation angle, but different scatter values.

In this study, we would like to test one possible factor that may limit the refinement of the cortical retinotopic map: the amount of sensory input. We explored this question in the binocular region of the mouse visual cortex. In this cortical area the same population of neurons receives input from both eyes. Only about 5% of neurons in this area are normally monocular [9]. Thus, there are two retinotopic maps in the same cortical area, both covering the same region of visual space. This system is well suited to address our question because the amount of sensory information coming into the network is readily manipulated by simply removing input from one eye (e.g., interocular injections of receptor blockers to silence the retina or surgical removal). Most importantly, using intrinsic signal optical imaging, a fundamental aspect of visual cortex organization, the retinotopic map, can be measured (Figure 1A). Fundamentally, retinotopic organization forms the basis for many higher order functions of visual cortex including orientation selectivity [10]. Therefore, in chronic imaging experiments in adult mice, we made quantitative measurements of the retinotopic maps before and after enucleation.

## Results

We used Fourier analysis of optically imaged intrinsic signals to map cortex responsiveness and isoelevation retinotopy (Figure 1A). We chronically imaged ipsilateral eye maps in adult mice in which cranial windows were implanted over the visual cortex [11]. After imaging every other day for six days, all mice were monocularly enucleated contralaterally and then followed for another 22 days, for a total of 28 days. From the retinotopic maps, we took three measurements: magnitude of response to visual stimulation, area of responsive cortex, and map scatter.

Map scatter is a metric that has been used to measure the smoothness of retinotopic maps [2], [12]–[13]. Map scatter is defined as the difference, in degrees of elevation or azimuth in visual space, between the receptive field center of one pixel and the average of its surrounding pixels in a 5×5 box (Figure 1B). If the retinotopic map is perfectly smooth, the average map scatter value will be near zero. High map scatter values indicate that the portion of cortex imaged in the pixel exhibits a receptive field that is not closely related to the receptive fields of adjacent areas. Low map scatter maps also exhibit more smoothly varying receptive fields across the cortex when measured using single unit electrophysiology [1], [14]. In development, map scatter values start out high in young mice and quickly asymptote to low values as the visual system matures [2].

**Figure 2:**
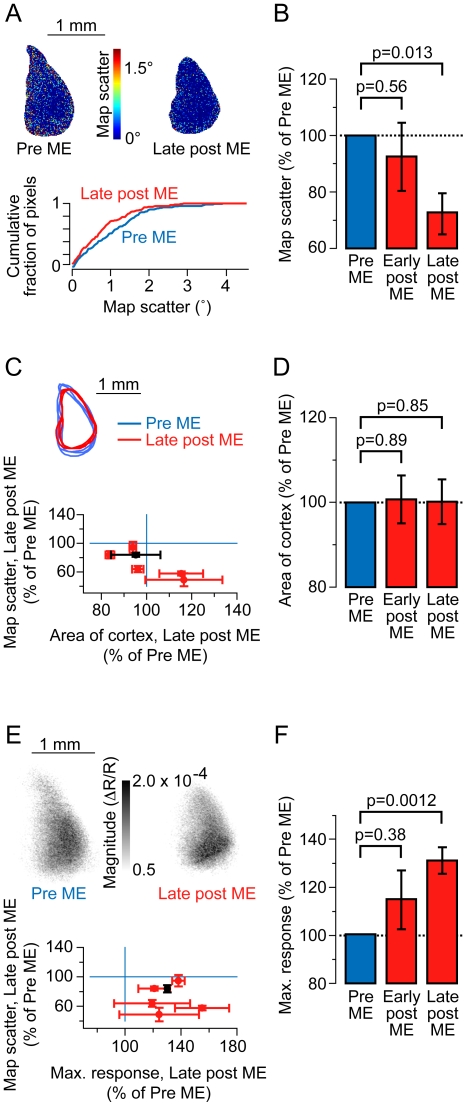
Chronic *in vivo* imaging in adult mice reveals improvements in ipsilateral retinotopic maps after monocular enucleation (ME) of the contralateral eye. (A, upper panel) Example scatter maps from the same animal show a reduction in scatter values after ME. (A, lower panel) Cumulative histogram of the two maps shown in the upper panel shows a leftward shift after ME, indicating more low scatter pixels in the retinotopic map. (B) Group data show that scatter values do not decrease immediately, but decrease 30% during the third week after ME (late post ME). (C, upper panel) Outlines of responsive cortex for the three pre ME time points and the three late post ME time points in the same animal overlap each other indicating little change in the area or location of responsive cortex. The outlines were aligned using vascular images. (C, lower panel) Change in scatter is plotted versus the change in area of responsive cortex showing that scatter values consistently decrease (points lie below the horizontal line) while no trend in area changes is observed (points evenly distributed on either side of the vertical line). Data from the example animal is colored black. (D) Group data show that the area of responsive cortex does not change after ME. (E, upper panel) Example magnitude maps show responsiveness before ME and during the third week after ME (late post ME). (E, lower panel) Change in scatter is plotted versus the change in maximum response magnitude showing that scatter values consistently decrease (points lie below the horizontal line) while maximum response magnitude consistently increases (points lie to the right of the vertical line). Data from the example animal is colored black. (F) Group data show that maximum response magnitude increases 30% during the third week after ME (late post ME). Reported p-values were computed using a paired, two-tailed t-test.

Time points were pooled into three epochs: pre ME data was obtained by averaging measurements taken over the six days preceding ME, early post ME consists of measurements taken over the two weeks following ME, and late post ME time point consists of measurements taken in the third week after ME. In mice that had their contralateral eye removed, the map scatter of ipsilateral eye retinotopic maps significantly decreased (Figure 2A). These changes were significant when the pre ME period were compared to the late post ME period (72.2±7.3%, control = 100%; p = 0.013, paired, two-tailed t-test; Figure 2B). This improvement was not accompanied by an increase in the area of responsive cortex (100.1±5.3%; p = 0.85, paired, two-tailed t-test; Figure 2C, D). The maximal amplitude of intrinsic signals evoked by ipsilateral eye stimulation increased (Figure 2E) as expected from prior chronic electrode recordings [15] (131.3±5.5%; p = 0.0012, paired, two-tailed t-test; Figure 2F).

Prior to monocular enucleation, ipsilateral eye retinotopic maps were obtained by placing an opaque material just in front of the contralateral eye during the imaging session. In this paradigm, the contralateral eye still exhibits unpatterned, spontaneous activity. To determine whether the absence of this activity could alone account for the observed change, we analyzed maps directly after enucleation. No significant change was seen immediately following enucleation (p = 0.56, Figure 2B), therefore the decreased scatter was not caused simply by a loss of contralateral vision or the contralateral eye per se, but by a slower process of cortical refinement following enucleation.

**Figure 3:**
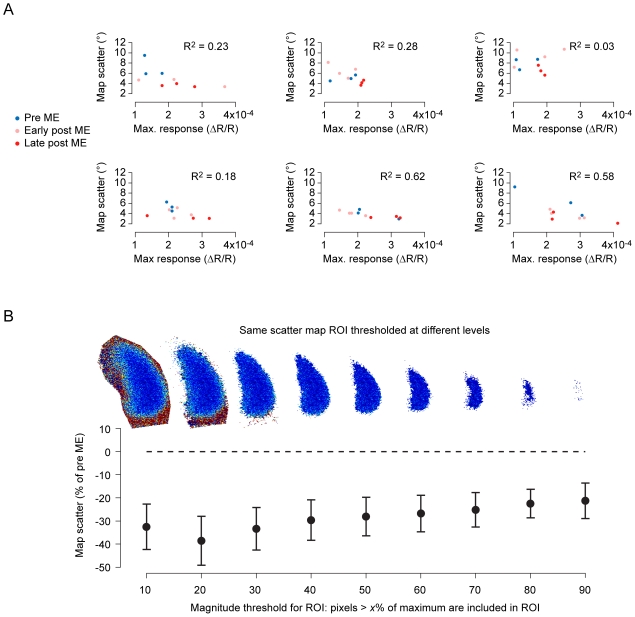
Map scatter and response magnitude are significantly independent measures and a threshold used in analysis does not affect the results of present study. (A) For six animals, map scatter measurements are plotted against response magnitude. Each marker is a different time point in the color-coded period. The Pearson's R^2^ correlation measurement for each animal is shown above each scatter plot. There was no systematic, strong correlation observed between response magnitude and map scatter, thus indicating a significant amount of independence in these two measures. (B) Magnitude maps were thresholded at a level equal to 60% of the maximum in order to define a region of interest for analysis of scatter maps. The results of the current study have been recomputed for various alternative threshold levels. Example scatter maps with the indicated threshold applied are shown above a plot of reduction in map scatter after ME versus threshold used in the analysis.

To ensure that map scatter and response magnitude are significantly independent measures, we looked for correlations between response amplitude and map scatter (Figure 3A). We found no consistent relationship, thus confirming there is a significant amount of independence in these two measurements. Finally, to test the robustness of the results, we repeated the analysis using an array of different threshold levels for defining the region of interest (Figure 3B). We found that the results were not sensitive to this parameter of the analysis.

## Discussion

These measurements show that binocular visual cortex reconfigured its circuitry to create lower scatter retinotopic maps of the ipsilateral eye after removing input from the contralateral eye. Notably, this refinement was never accompanied by an expansion in the area of cortex responding to ipsilateral eye stimulation. Thus it is unlikely that additional neurons were recruited to expand the circuitry into areas of cortex that previously did not respond to ipsilateral eye stimulation. Instead, our results suggest that plastic circuit reorganization within a fixed population of neurons underlies the improvement in the map. Therefore, in these experiments, we observed an increase in refinement of the retinotopic map in adult animals, beyond the normal, asymptotic level. This was enabled by removing the contralateral eye and thus is consistent with the hypothesis that the circuitry itself limits the refinement of the retinotopic map.

Prior to these experiments, there was no evidence that each eye's retinotopic map in binocular cortex were sub-maximally refined. That is, there was no reason to believe that visual cortex did not intermingle two sets of inputs such that each was maximally refined. In fact, Hebbian mechanisms could be expected to support such a development, with neurons receiving input from both eyes, each encoding for the same region of visual space, and thus the maximal level of refinement would have been limited by the quality of retinal input or other mechanisms. However, our results are inconsistent with the hypothesis that the quality of the sensory input limits the refinement since that variable was not altered. One possible explanation for the enhanced refinement is that synapses are recruited by the remaining ipsilateral eye to improve that eye's retinotopic map. This concept of a fixed amount of synaptic resources that can be plastically repurposed fits with results showing that dendritic spines are gained and lost in an experience-dependent manner in adult cortex with only modest changes in density [11], [16]–[17].

Interestingly, in these adult experiments monocular enucleation did not increase the area of responsive cortex. This is in contrast to critical period monocular enucleation, which results in an expansion of responsive cortex area [18], possibly because neocortical circuitry is more amenable to large-scale reconfiguration during the critical period. In the adult animals used in the current study, since most neurons in binocular mouse visual cortex respond to stimulation from either eye [9], perhaps no additional neurons were recruited to generate the further refined retinotopic map. In this case, synapses may be reconfigured among already binocular neurons to process visual input from the ipsilateral eye. The increased synaptic drive to the cortex then increased the overall response to ipsilateral eye visual stimulation, as detected in our experiments. The limiting factor, then, would solely be synaptic, rather than neuronal. Although this scenario is plausible, we cannot rule out the possibility that relatively unresponsive neurons [19] in binocular cortex became responsive after monocular enucleation and thus effectively increased the pool of responsive neurons. Future studies should explore changes in single neuron response properties such as receptive field size.

Notably, psychophysical studies in humans [4]–[6] and rodents [7]–[8] show that visual acuity increases when the function of one eye is lost. The mechanism of this phenomenon is unknown and could be a result of changes in higher level processing, rather than changes in primary visual cortex. It is possible that the changes reported above may contribute to the psychophysical effects. Monocular deprivation has been shown to increase acuity in visual cortex as measured by visually evoked potentials [20]. However, further studies are required to test the link between the two phenomena.

In summary, further refinement of retinotopic maps is possible in adult animals when input from the contralateral eye is removed. This supernormal refinement is accompanied by an increase in response magnitude, but not in the area of responsive cortex. These results are consistent with the hypothesis that visual cortical circuitry is normally refined to the maximum capacity, or saturation.

## Materials and Methods

### Optical imaging

All procedures involving the handling and use of mice for these experiments were approved by the University of California Los Angeles Office for Protection of Research Subjects and the Chancellor's Animal Research Committee. Six mice (C57/bl6, 2–6 months old; Taconic, Charles River) were imaged chronically for this study. Mice were anesthetized with halothane (5% for induction, 1–2% for maintenance) and mounted in a stereotaxic frame. The eyes were covered with silicon oil. The existing optical window was gently cleaned with water, if necessary. The preparation was illuminated with 700 nm light and imaged with a tandem lens macroscope defocused 600 µm into the brain. Images were acquired with a 12-bit CCD camera (Dalsa 1M30), frame grabber (Matrox Meteor II/Dig), and custom software. The visual stimulus was a white horizontal bar, 1–2 degrees in height, which drifted up or down at 0.125 Hz on a black background. An eight minute long movie was taken for each direction and each eye, making for four movies total. Acquisition was at 30 frames per second, and the 12 bit frames were binned in software 4x temporally and 2×2 spatially, resulting in 16 bit image files. From movies of these 16 bit files, Fourier analysis of each pixel column generated maps of magnitude and phase at 0.125 Hz. Scatter was computed on phase maps that were corrected for hemodynamic delay using time reversal. The region of interest was defined using the magnitude map after it had been smoothed using a 5×5 Gaussian filter. All pixels that had magnitude values >60% of the peak level were considered responsive cortex and included for analysis, however cutoff levels between 10% and 90% yielded the same results. The phase value for each pixel in the masked phase map was compared to the average of its neighbors in a 5×5 box (4444 µm^2^ of cortex), and the average difference for each map was calculated. In practice, this method is capable of detecting retinotopic maps even when response magnitude is weak (Cang, Kaneko, et al., 2005; Smith and Trachtenberg 2007). Maximum magnitude values were taken from unfiltered magnitude maps.

### Monocular enucleation (ME)

Mice were anesthetized with ketamine/xylazine or isoflurane. The conjectiva was trimmed, the eyeball displaced from the socket, and the optic nerve tied off with 6–0 suture. The optic nerve was then cut and the eyeball was removed. Antibiotic ointment was placed in the orbit and the eyelid was sutured closed. Mice received daily injections of carprofen analgesic for two days after surgery.

### Chronic adult imaging

Adult experiments were initiated when the animals were at least three months old. Mice were anesthetized with ketamine/xylazine and a portion of the scalp was removed. A dental drill was used to remove a 2–3 mm diameter portion of skull. A glass coverslip was placed on the dura and secured with cyanoacrylate glue. The wound margins and edges of the coverslip were covered with dental acrylic. The animal was allowed to recover for at least a week with daily injections of carprofen analgesic for the first five days. ME surgery, as described above, took place in these animals after six days of baseline imaging.

### Statistics

The paired and unpaired two-tailed t-tests were used to compute significance. The paired test was used for comparing pre-ME and post-ME measurements on the same animals. All error bars indicate S.E.M.
